# TREC and KREC Levels as a Predictors of Lymphocyte Subpopulations Measured by Flow Cytometry

**DOI:** 10.3389/fphys.2018.01877

**Published:** 2019-01-21

**Authors:** Ilya Korsunskiy, Oleg Blyuss, Maria Gordukova, Nataliia Davydova, Susanna Gordleeva, Robert Molchanov, Alan Asmanov, Dmitrii Peshko, Nataliia Zinovieva, Sergey Zimin, Vladimir Lazarev, Aminat Salpagarova, Maxim Filipenko, Ivan Kozlov, Andrey Prodeus, Anatoliy Korsunskiy, Peter Hsu, Daniel Munblit

**Affiliations:** ^1^Speransky Children’s Hospital, Moscow, Russia; ^2^Department of Paediatrics, Sechenov University, Moscow, Russia; ^3^Dmitry Rogachev National Research Center of Pediatric Hematology, Oncology and Immunology, Moscow, Russia; ^4^Wolfson Institute of Preventive Medicine, Queen Mary University of London, London, United Kingdom; ^5^Lobachevsky State University of Nizhny Novgorod, Nizhny Novgorod, Russia; ^6^State Institution “Dnipropetrovsk Medical Academy of the Ministry of Health of Ukraine”, Dnipro, Ukraine; ^7^The Research and Clinical Institute for Pediatrics named after Academician Yuri Veltischev of the Pirogov Russian National Research Medical University, Moscow, Russia; ^8^Pharmacogenomic Laboratory, Institute of Chemical Biology and Fundamental Medicine SB RAS, Novosibirsk, Russia; ^9^Immanuel Kant Baltic Federal University, Kaliningrad, Russia; ^10^Allergy and Immunology, The Kids Research Institute, The Children’s Hospital at Westmead, Sydney, NSW, Australia; ^11^The In-VIVO Global Network, An Affiliate of the World Universities Network, New York, NY, United States; ^12^Department of Paediatrics, Imperial College London, London, United Kingdom; ^13^Solov’ev Research and Clinical Center for Neuropsychiatry, Moscow, Russia

**Keywords:** TREC, KREC, primary immune deficiency, PID, flow cytometry, lymphocyte subpopulations, immunoglobulins

## Abstract

Primary immunodeficiency diseases (PID) is a heterogeneous group of disorders caused by genetic defects of the immune system, which manifests clinically as recurrent infections, autoimmune diseases, or malignancies. Early detection of other PID remains a challenge, particularly in older children due to milder and less specific symptoms, a low level of clinician PID awareness and poor provision of hospital laboratories with appropriate devices. T-cell recombination excision circles (TREC) and kappa-deleting element recombination circle (KREC) in a dried blood spot and in peripheral blood using real-time polymerase chain reaction (PCR) are used as a tool for severe combined immune deficiency but not in PID. They represent an attractive and cheap target for a more extensive use in clinical practice. This study aimed to assess TREC/KREC correspondence with lymphocyte subpopulations, measured by flow cytometry and evaluate correlations between TREC/KREC, lymphocyte subpopulations and immunoglobulins. We carried out analysis of data from children assessed by clinical immunologists at Speransky Children’s Hospital, Moscow, Russia with suspected immunodeficiencies between May 2013 and August 2016. Peripheral blood samples were sent for TREC/KREC, flow cytometry (CD3, CD4, CD8, and CD19), IgA, IgM, and IgG analysis. A total of 839 samples were analyzed for using TREC assay and flow cytometry and 931 KREC/flow cytometry. TREC demonstrated an AUC of 0.73 (95% CI 0.70–0.76) for CD3, 0.74 (95% CI 0.71–0.77) for CD4 and 0.67 (95% CI 0.63–0.70) for CD8, respectively, while KREC demonstrated an AUC of 0.72 (95% CI 0.69–0.76) for CD19. Moderate correlation was found between the levels of TREC and CD4 (*r* = 0.55, *p* < 0.01) and KREC with CD19 (*r* = 0.56, *p* < 0.01). In this study, promising prediction models were tested. We found that TREC and KREC are able to moderately detect abnormal levels of individual lymphocyte subpopulations. Future research should assess associations between TREC/KREC and other lymphocyte subpopulations and approach TREC/KREC use in PID diagnosis.

## Introduction

Primary immunodeficiency diseases (PID) is a heterogeneous group of disorders caused by genetic defects of the immune system, which manifests clinically as recurrent infections, autoimmune diseases or malignancies. Severe forms of PID – Severe Combined Immune Deficiency (SCID) – are associated with inherited lack of cellular and humoral immunity caused by mutations in various genes (Chan and Puck, 2005) and associated with a significant mortality rates in the first 2 years of life (Dvorak et al., 2013; Yao et al., 2013).

Severe combined immune deficiency can be detected by T-cell receptor excision circles (TRECs) measurement in a dried blood spot using real-time polymerase chain reaction (PCR) (Chan and Puck, 2005). TREC measurement became a part of neonatal screening in the United States and some other countries (Verbsky et al., 2012; Kwan et al., 2013). Despite great predictive value TREC can detect T-cells production defects, but not isolated B-cell defects. Some experts suggested that kappa-deleting element recombination circle (KREC) may add value in PID diagnosis (Nakagawa et al., 2011) and multiplex techniques for simultaneous quantitation of TREC/KREC were piloted (Borte et al., 2012).

Outside of neonatal screening, TREC/KREC measurement is not commonly used in routine clinical practice, with flow cytometry being a traditional, but more expensive diagnostic technique for PID detection, when compared with the PCR (Puck and SCID Newborn Screening Working Group, 2007). It requires a significant amount of training and not readily available in many developing countries. TREC and KREC assessment both in PID diagnosis and in therapy monitoring represent great potential (Serana et al., 2013).

TREC and KREC predictive ability in SCID has been extensively studied, but not much research was done in relation to physiological aspects of relationships between TREC/KREC and lymphocyte subpopulations. In this pilot study we assessed correlations between TREC/KREC levels, lymphocyte subpopulations and immunoglobulins and evaluated TREC/KREC ability to predict reduced levels of lymphocyte subpopulations.

## Materials and Methods

### Study Setting, Eligibility Criteria, and Ethics

We carried out a retrospective analysis of data from all children assessed by clinical immunologists at Speransky Children’s Hospital, Moscow, Russia with suspected immunodeficiencies between May 2013 and August 2016. The diagnosis of different types of PID was based on IUIS Phenotypic Classification for Primary Immunodeficiencies (Bousfiha et al., 2015). The investigations and sample collection have been conducted following ethical approval by the Speransky Children’s Hospital Ethics Committee. Parental written consent was obtained for all participants as a part of routine procedure at Speransky Children’s Hospital. Parents/guardians were informed of the procedures in lay terms.

### Sample Analysis

Peripheral blood samples were taken by venipuncture during morning hours, aliquoted and sent for CBC, flow cytometry, immunoglobulins (IgA, IgM, and IgG) and TREC/KREC analysis. All blood samples were EDTA-anticoagulated and analyzed on the same day of the collection in order to avoid cellular death.

#### Immunophenotyping

Three-Four color flow cytometric immunophenotyping with directly labeled monoclonal antibodies was used to determine the following immune cell subsets: CD3, CD4, CD8, CD19 following manufacturer’s protocol. In brief, 50 μl aliquots of blood were incubated for 15 min in the dark at room temperature with combinations of optimally titrated fluorochrome-conjugated monoclonal antibodies. After surface staining, erythrocytes were lysed using 1 ml of BD FACSLysing Solution, diluted according to manufacturer’s instructions. Remaining cells were washed twice and suspected in CELL WASH buffer for further analysis for a FACS Canto II flow cytometer using FACSDiva v7.0 software (Becton Dickinson). Cell suspension for staining of naïve and memory B-lymphocytes were prepared as described by Ferry et al. (2005). Briefly, 300 μl blood aliquots were washed three times using CELL WASH buffer (Becton Dickinson) supplemented with 2% bovine serum albumin to eliminate any cell-bound antibodies non-specifically inhibiting the staining effect.

Acquisition was run until 10000–50000 events were detected. First the viable part of the sample was selected by physical gating based on forward scatter (FS) and side scatter (SS); the lymphocyte population was identified by the low forward and side scatter and checked for purity by the positivity for CD45. Then the different lymphocyte subpopulations were identified by immunophenotype markers. At least 5000 events from B-lymphocyte gate set based on CD19 expression and side scatter characteristics were acquired.

The total leucocytes count and differential was measured with Advia 2120i hematology analyzer (Siemens). The absolute size of each lymphocyte subpopulation was calculated by multiplying the relative size of the lymphocyte subpopulation and the absolute lymphocyte count.

### Immunoglobulins Assay

Immunoglobulin levels were assessed by immunoturbidimetry method using biochemical analyzer Architect C8000 (Abbott, United States, Abbott kits) in accordance with manufacturers’ protocol.

#### TREC and KREC Assay

TREC and KREC assay was performed using real-time PCR with fluorescent hybridization probes with reagents for TREC/KREC assay T&B PCR kit (ABV-test, Russia) (Deripapa et al., 2017) in whole blood and dry blood stain DNAs.

The TREC/KREC levels were assayed in whole blood samples as described previously (Sottini et al., 2010; Deripapa et al., 2017). In brief, DNA was extracted from 100 μl EDTA anticoagulated whole blood by using RIBO-prep nucleic acid extraction kit (Amplisense^®^, Russia). The Real-time qPCR was performed by using CFX 96 Real-Time PCR System (Bio Rad, United States).

### Statistical Analysis

Shapiro-Wilk test has been used to assess the normality of the distribution of variables analyzed in this paper. Since the null hypothesis about the normality was rejected, Spearmen correlation coefficient was used to assess the strength of the correlation between the variables. Sensitivity, specificity and their 95% confidence intervals were computed with stratified bootstrap replicates (Carpenter and Bithell, 2000). Area under Receiver Operating Characteristic (ROC) -curve (AUC) calculation was followed by 95% confidence interval as suggested by DeLong (DeLong et al., 1988). To account for non-linear trend, level of TREC, KREC, and lymphocyte subpopulations were substituted by the ratio of their concentrations to corresponding reference values for a given patient age.

Results were considered statistically significant if *p*-value was smaller than 0.05. All calculations were done using R package version 3.4.1.

#### TREC/KREC and Lymphocyte Subpopulations

Primary analysis consisted of two stages.

At stage 1 we assessed 4 paired relationships between the levels of TREC with CD3, CD4, CD8, and KREC with CD19. These were presented as a proportion of patients with normal levels of one of the paired variable among patients with normal levels of another immunological marker.

We also assessed correlations between TREC/KREC and lymphocyte subpopulations.

At stage 2 we assessed ability of TREC and KREC to predict abnormality in lymphocyte subpopulation levels. Using ROC-analysis the predictivity of TREC, KREC and their combination was tested providing: (a) the sensitivity (proportion detected of those with lower lymphocyte subpopulation levels) at a fixed specificity (proportion of controls correctly detected not to have reduced lymphocyte subpopulation levels) and (b) AUC.

## Results

### Study Population

The data was extracted from the clinical notes and laboratory database of Speransky Children’s Hospital. Out of all 3055 patients requiring flow cytometry within the given period of time, due to financial restrictions, a total of 839 samples were analyzed using flow cytometry and TREC assay and 931 samples were analyzed using flow cytometry and KREC assay and were included into the statistical analysis. Data on TREC/KREC levels of 2050 children were unavailable and were not evaluated further. Data on clinical diagnosis was available in 471 participant and presented in Table 1.

**Table 1:** Characteristics of study participants.

PID type	ICD-10 (number of patients)	Age	Gender	
			Male	Female
Type I	D81 (17)	0–12 months	9	5
Immunodeficiencies affecting cellular and humoral immunity		1–6 years	2	0
		6–12 years	1	0
		12–18 years	0	0
Type II	D82 (13)	0–12 months	7	9
CID with associated or syndromic features	D82.1 (39)	1–6 years	26	17
	D82.4 (5)	6–12 years	10	6
	D84.8 (15)	12–18 years	4	3
	G11.3 (8)			
Type III	D80.0 (4)	0–12 months	6	2
Predominantly antibody deficiencies	D80.1 (47)	1–6 years	21	17
	D80.2 (34)	6–12 years	28	21
	D80.3 (24)	12–18 years	38	13
	D80.4 (1)			
	D80.5 (4)			
	D83 (34)			
Healthy children		0–12 months	9	5
		1–6 years	41	48
		6–12 years	36	33
		12–18 years	25	29

*Provided codes are in accordance with International Classification of Diseases, 10th revision (ICD-10).*

### Comparison of Flow Cytometry Parameters With TREC and KREC

At first stage ability of TREC/KREC test to predict CD19, CD3, CD4 and CD8 flow cytometry results was assessed. We found that 667 out of 863 (77.3%) patients with normal KREC levels [as reported earlier (Gordukova et al., 2015)] had normal CD19, while 667 out of 682 (97.8%) individuals with normal CD19 had normal KREC.

In patients with normal TREC levels, 462 out of 548 (84.3%) had normal CD3, 440 out of 548 (80.3%) normal CD4, and 473 out of 548 (86.3%) normal CD8 counts. Individuals having normal levels of CD3, CD4, and CD8 had normal levels of TREC in 462/548 (84.3%), 440/548 (80.3%), and 473/548 (86.3%), respectively.

Values of TREC, KREC and lymphocyte subpopulations which were considered abnormally low for the purpose of this study are reported in Supplementary Table S1.

### TREC/KREC Ability to Predict Abnormal Levels in Lymphocyte Subpopulations

**FIGURE 1:**
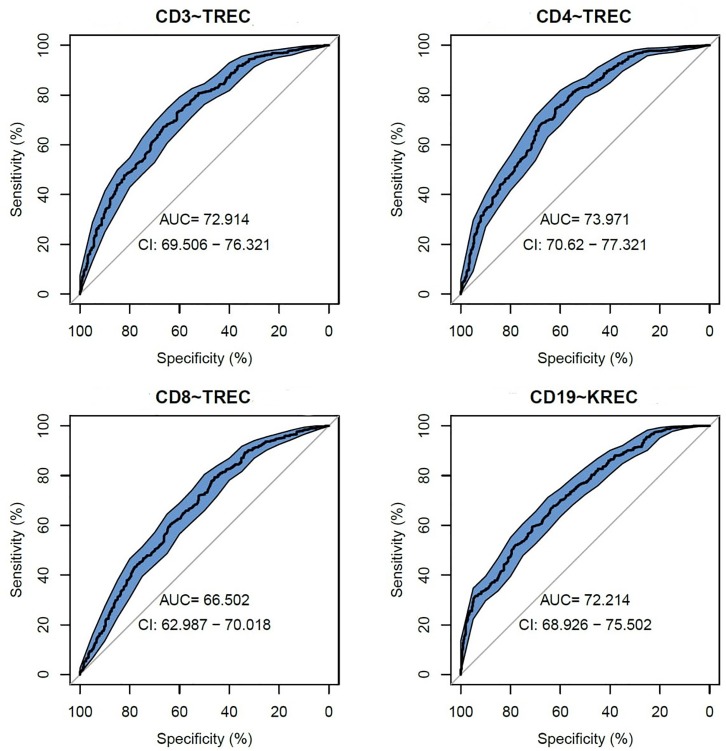
Receiver operating characteristic (ROC) curves for TREC and KREC for the ability to predict abnormal values of lymphocyte subpopulations (CD3, CD4, CD8, and CD19), (*n* = 931).

We assessed TREC/KREC ability to predict each lymphocyte subpopulation individually, using area under the curve (AUC), which are shown in Figure 1. TREC demonstrated an AUC of 0.73 (95% CI 0.70–0.76) for CD3, 0.74 (95% CI 0.71–0.77) for CD4 and 0.67 (95% CI 0.63–0.70) for CD8, respectively, while KREC demonstrated an AUC of 0.72 (95% CI 0.69–0.76) for CD19.

The following cutoff points of a probability showed the best prognostic accuracy with regards to sensitivity and specificity for TREC: 0.6 (67% for both, sensitivity, and specificity; Table 2), Youden’s index (*J*) = 32.9 in CD3; 0.55 (77 and 58%; Table 3), J = 35.1 in CD4; 0.5 (80 and 44%; Table 4), J = 24.6 in CD8, respectively. A cutoff point of a probability of 0.55 showed the best diagnostic accuracy with regards to sensitivity and specificity for KREC (67% and 64%; Table 5), J = 30.4 in predicting abnormal levels of CD19.

**Table 2:** Model performance for different cutoff points of the predicted probabilities for TREC with regards to CD3.

Cutoff (probability) (probability)	PPV (%)	NPV (%)	Sensitivity (%)	Specificity (%)	Youden index
0.5	69	76	97	17	13.7
0.55	76	58	81	50	31.3
**0.6**	**79**	**51**	**67**	**67**	**32.9**
0.65	81	46	52	78	29.2
0.7	85	44	41	86	27.1
0.75	86	41	31	90	21
0.8	88	39	23	94	17.4

*Optimum cut-off point based on maximum value of the J index is presented in bold.*

**Table 3:** Model performance for different cutoff points of the predicted probabilities for TREC with regards to CD4.

Cutoff (probability) (probability)	PPV (%)	NPV (%)	Sensitivity (%)	Specificity (%)	Youden index
0.5	72	76	94	34	27.9
**0.55**	**76**	**59**	**77**	**58**	**35.1**
0.6	79	51	61	71	32
0.65	81	47	48	80	27.9
0.7	83	44	36	87	23.6
0.75	87	42	28	93	20.7

*Optimum cut-off point based on maximum value of the J index is presented in bold.*

**Table 4:** Model performance for different cutoff points of the predicted probabilities for TREC with regards to CD8.

Cutoff (probability) (probability)	PPV (%)	NPV (%)	Sensitivity (%)	Specificity (%)	Youden index
**0.5**	**65**	**64**	**80**	**44**	**24.6**
0.55	69	52	48	73	21
0.6	72	48	28	86	14.1
0.65	70	46	18	90	8.4
0.7	72	45	11	95	5.3

*Optimum cut-off point based on maximum value of the J index is presented in bold.*

**Table 5:** Model performance for different cutoff points of the predicted probabilities for KREC with regards to CD19.

Cutoff (probability) (probability)	PPV (%)	NPV (%)	Sensitivity (%)	Specificity (%)	Youden index
0.5	71	58	79	48	26.9
**0.55**	**75**	**54**	**67**	**64**	**30.4**
0.6	77	50	55	74	28.2
0.65	80	47	45	82	26.6
0.7	82	46	38	86	23.9
0.75	87	45	33	92	24.5
0.8	91	44	26	96	21.8

*Optimum cut-off point based on maximum value of the J index is presented in bold.*

**FIGURE 2:**
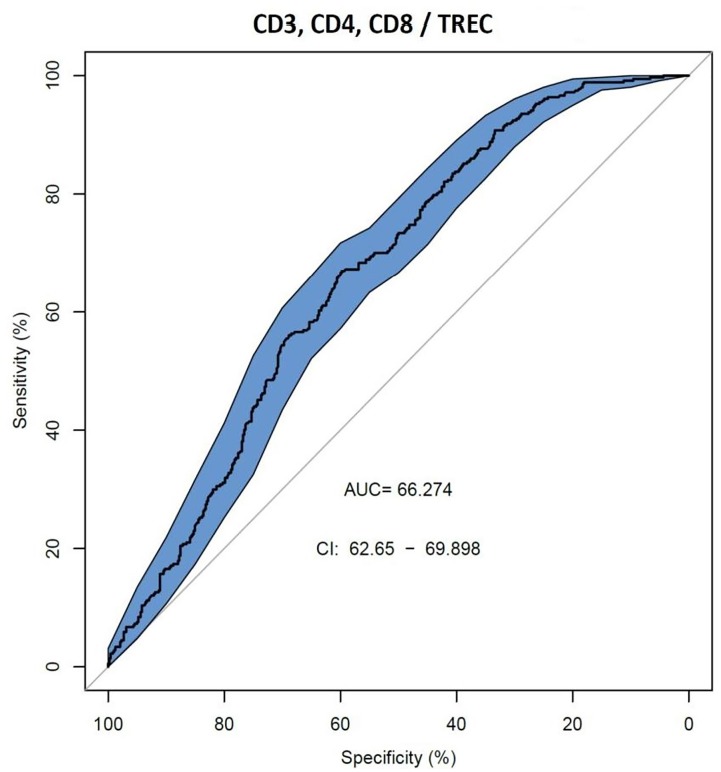
Receiver operating characteristic (ROC) curves for TREC for the ability to predict abnormal values of lymphocyte subpopulations (CD3, CD4, and CD8), (*n* = 839).

**FIGURE 3:**
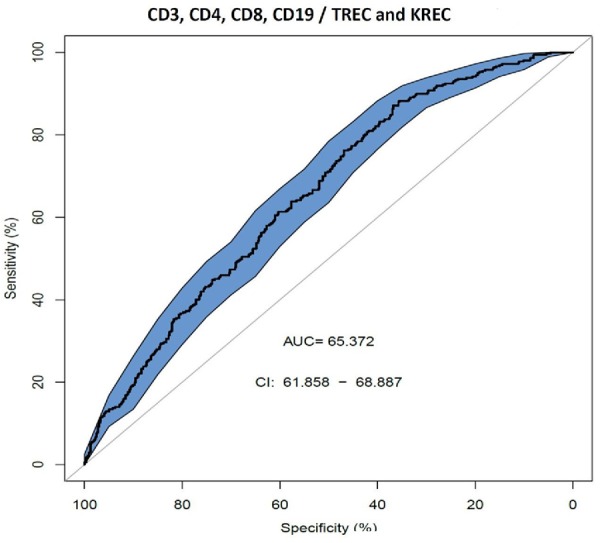
Receiver operating characteristic (ROC) curves for combination of TREC and KREC for the ability to predict abnormal values of lymphocyte subpopulations (CD3, CD4, CD8, and CD19), (*n* = 931).

We also assessed AUC for TREC ability to predict the reduced levels of CD3, CD4 and CD8 (Figure 2), and a combination of TREC and KREC (Figure 3) to predict the reduced levels of all lymphocyte subpopulations analyzed. TREC demonstrated an AUC of 0.66 (95% CI 0.63–0.70) while a combination of TREC and KREC resulted in an AUC of 0.65 (95% CI 0.62–0.69). A cutoff point of a probability of 0.4 showed the best diagnostic accuracy with regards to sensitivity and specificity for TREC (59% and 65%; Table 6), J = 23 and 0.35 for a combination of TREC and KREC (61 and 59%, respectively; Table 7), J = 20.2.

**Table 6:** Model performance for different cutoff points of the predicted probabilities for TREC with regards to CD3,4, and 8.

Cutoff (probability) (probability)	PPV (%)	NPV (%)	Sensitivity (%)	Specificity (%)	Youden index
**0.4**	**55**	**68**	**59**	**65**	**23**
0.45	55	61	28	83	10.7
0.5	56	59	17	90	6.7

*Optimum cut-off point based on maximum value of the J index is presented in bold.*

**Table 7:** Model performance for different cutoff points of the predicted probabilities for combined TREC and KREC with regards o all lymphocyte subpopulations measured.

Cutoff (probability) (probability)	PPV (%)	NPV (%)	Sensitivity (%)	Specificity (%)	Youden index
0.3	43	85	93	24	17.3
**0.35**	**48**	**71**	**61**	**59**	**20.2**
0.4	52	68	43	75	18.2
0.45	54	66	28	86	13
0.55	65	64	13	96	8.5

*Optimum cut-off point based on maximum value of the J index is presented in bold.*

### Correlation Between TREC/KREC and Lymphocyte Subpopulations

We observed a moderate correlation (Table 8) between the levels of TREC and CD4 (*r* = 0.55, *p* < 0.01) and KREC with CD19 (*r* = 0.56, *p* < 0.01). Moderate to low correlation was found between TREC with CD19, CD3 and lymphocytes (*r* between 0.28 and 0.46, *p* < 0.01) and KREC with CD4 and lymphocytes (*r* = 0.33, *p* < 0.01). Neither TRECs nor KRECs levels correlated with the concentration of immunoglobulins (IgA, IgG).

**Table 8:** Heatmap of correlation between TRECs/KRECs level with immunoglobulins, CDs and blood cells.

	ley	lym	IgG	IgA	IgM	CD4	CD8	CD3	CD19
**TREC**	0.09^∗∗^	0.41^∗∗^	−0.08^∗^	−0.11^∗∗^	0.002^NS^	0.55^∗∗^	0.28^∗∗^	0.46^∗∗^	0.34^∗∗^
**KREC**	0.11^∗∗^	0.33^∗∗^	−0.17^∗∗^	−0.15^∗∗^	−0.10^∗∗^	0.33^∗∗^	0.12^∗∗^	0.25^∗∗^	0.56^∗∗^

*More intense green color means stronger positive correlations, while more intense red color means stronger negative correlation. ^∗^p < 0.05; ^∗∗^p < 0.01; NS, statistically not significant result.*

## Discussion

In this study, we assessed associations between TREC/KREC and lymphocyte subpopulations. TREC and KREC models showed good ability to predict abnormal levels of certain lymphocyte subpopulations and modest correlations between TREC and CD4, KREC and CD19 were found.

PID is a large group of disorders encompassing a few hundred various conditions affecting development and/or functioning of the immune system (Picard et al., 2015). Flow cytometry is a sensitive and important tool in immune system functioning evaluation and PID diagnosis (Kanegane et al., 2018), however, it is expensive, not easily available and complexity of the method requires appropriate training. TREC and KREC may represent a cheaper alternative and/or add value to PID diagnosis and screening. Low cost methodology can be used in small laboratories and rural settings, where complex and expensive tools are unavailable, to provide access to primary PID evaluation. TREC/KREC evaluation may also serve as a prerequisite to flow cytometry.

We found significant correlations between the levels of TREC and lymphocyte subpopulations with the strongest correlations were observed for TREC/CD3, TREC/CD4. This finding is consistent with previous reports (Mensen et al., 2013; Gul et al., 2015), suggesting that low TREC levels correlate with low values of CD3+ and CD4+. The observed positive correlation could be attributed to TRECs being a direct marker for thymic output (Ravkov et al., 2017). We also observed a statistically significant moderate KREC levels correlation with CD19 levels and we are not aware of other studies reporting this finding, however correlation is plausible as both KREC and CD19 are linked with B-lymphocytes.

When proportion of patients with both normal TREC/KREC and lymphocyte subpopulations was assessed, we found that almost every individual with CD19 within the reference range had normal KREC levels. Most of individuals (80–85%) with CD3, CD4 and CD8 within the reference range had normal TREC levels. Given moderate correlations between TREC/KREC and lymphocyte subpopulations and promising proportion results, we expected a decent ability of TREC and KREC predictive models with regards to lymphocyte subpopulations abnormal levels. Positive predictive values for TREC ability to predict abnormal levels of CD3 and CD4, and KREC abnormal levels of CD19 varied between 75 and 79%, when optimum cut-off point was selected based on maximum value of the *J* index. TREC ability to predict abnormal level of CD8, in contrast, was much lower and reached a PPV of 65% only. This result was not surprising as negligible correlation between TREC and CD8 levels was detected.

Neither TREC, nor a combination of TREC and KREC reached impressive AUC values when predictivity of cumulative lymphocyte subpopulations was modeled. A cut-off points of a probability of 0.4 for TREC and 0.35 for a combination of TREC and KREC showed the best diagnostic accuracy according to Youden’s index but positive predictive value of the models was very low. We may speculate that lack of individual and multiple correlations between TREC and CD8; KREC and CD3, CD8, may explain lack of consistency in the model performance, when predictivity in relation to cumulative lymphocyte subpopulations was tested.

Models showed promising ability of TREC to predict abnormal levels of CD3 and CD4, and KREC abnormal levels of CD19. Although combined use of TREC and KREC did not result in good predictivity when cumulative lymphocyte subpopulations were assessed, further research may improve predictive ability, adding other subpopulations, such as naïve B-lymphocytes CD19+CD27-IgD+, recent thymic emigrants (RTE) and CD31+CD45RA+ T-lymphocytes. PID is a very heterogenous group of diseases, and TREC/KREC predictive abilities should be further tested in individuals with separate PID conditions. Future research should also target investigation of TREC/KREC diagnostic abilities in PID patients, which is an existing unmet need.

## Author Contributions

IK, MF, IK, AP, AK, and DM conceived and designed the experiments and study analysis. MG and ND performed the experiments. IK, NZ, SZ, AS, and DP collected, extracted, and sorted the data. OB and RM analyzed the data. VL and SG reviewed available evidence on the matter. AA, DP, IK, PH, and DM wrote the manuscript.

## Conflict of Interest Statement

MG, MF, and IK are board members for MG, MF, AP, and IK has a patent with ABV-test. The remaining authors declare that the research was conducted in the absence of any commercial or financial relationships that could be construed as a potential conflict of interest.
