# Supplementary material for: TREC and KREC Levels as a Predictors of Lymphocyte Subpopulations Measured by Flow Cytometry
Source: Front Physiol. 2019 Jan 21;9:1877. doi: 10.3389/fphys.2018.01877 (PMC6348265; doi:10.3389/fphys.2018.01877)
Supplement: Supplementary file 1 [file Table_1.DOCX]

| Lymphocyte subpopulations | | | | |
| --- | --- | --- | --- | --- |
| Age | CD3 | CD4 | CD8 | CD19 |
| 1-4 months | 2070 | 1460 | 650 | 500 |
| 5-12 months | 2280 | 1690 | 720 | 500 |
| 1-2 years | 1460 | 1020 | 570 | 500 |
| 2-6 years | 1610 | 900 | 630 | 700 |
| 6-12 years | 1400 | 700 | 600 | 300 |
| 12-18 years | 1400 | 700 | 600 | 300 |
| TREC and KREC | | | | |
|  | TREC | | KREC | |
| 0-11 months | 120000 | | 1000 | |
| 1-6 years | 14000 | | 1000 | |
| 7-12 years | 7300 | | 1000 | |
| 13-18 years | 2300 | | 1000 | |

**Table S1** Levels of TREC, KREC and lymphocyte subpopulations which were considered abnormal. Lymphocyte subpopulations concentrations are given in absolute count (n x 10^6^/L).
